# Cost determinants among adults hospitalized with respiratory syncytial virus in the United States, 2017–2019

**DOI:** 10.1111/irv.12912

**Published:** 2021-10-03

**Authors:** Yoonyoung Choi, Alexandra Hill‐Ricciuti, Angela R. Branche, William D. Sieling, Lisa Saiman, Edward E. Walsh, Matthew Phillips, Ann R. Falsey, Lyn Finelli

**Affiliations:** ^1^ Center for Observational and Real‐World Evidence, Merck & Co., Inc. Kenilworth New Jersey USA; ^2^ Department of Pediatrics Columbia University Irving Medical Center New York New York USA; ^3^ Department of Medicine, Division of Infectious Diseases University of Rochester Rochester New York USA; ^4^ Department of Medicine University of Minnesota Medical School Duluth Campus Duluth Minnesota USA; ^5^ Department of Infection Prevention & Control NewYork‐Presbyterian Hospital New York New York USA; ^6^ Rochester General Hospital Rochester New York USA

**Keywords:** active surveillance, adults, cost, predictors, RSV‐associated hospitalization

## Abstract

**Background:**

Respiratory syncytial virus (RSV) infections are common in adults, but data describing the cost of RSV‐associated hospitalization are lacking due to inconsistency in diagnostic coding and incomplete case ascertainment. We evaluated costs of RSV‐associated hospitalization in adult patients with laboratory‐confirmed, community‐onset RSV.

**Methods:**

We included adults ≥ 18 years of age admitted to three hospital systems in New York during two RSV seasons who were RSV‐positive by polymerase chain reaction (PCR) and had more than or equal to two acute respiratory infection symptoms or exacerbation of underlying cardiopulmonary disease. We abstracted costs from hospital finance systems or converted hospital charges to cost using cost‐charge ratios. We converted cost into 2020 US dollars and extrapolated to the United States. We used a generalized linear model to determine predictors of hospitalization cost, stratified by admission to intensive care units (ICU).

**Results:**

Cost data were available for 79% (601/756) of eligible patients. The mean total cost of hospitalization was $8403 (CI_95_ $7240–$9741). The highest costs were those attributed to ICU services $7885 (CI_95_ $5877–$10,240), whereas the lowest were radiology $324 (CI_95_ $275–$376). Other than longer length of stay, predictors of higher cost included having chronic liver disease (odds ratio [OR] 1.38 [CI_95_ 1.05–1.80]) for patients without ICU admission and antibiotic use (OR 1.49 [CI_95_ 1.10–2.03]) for patients with ICU admission. The annual US cost was estimated to be $1.2 (CI_95_ 0.9–1.4) billion.

**Conclusion:**

The economic burden of RSV hospitalization of adults ≥ 18 years of age in the United States is substantial. RSV vaccine programs may be useful in reducing this economic burden.

## 1 INTRODUCTION

Respiratory syncytial virus (RSV) causes upper and lower respiratory tract infections, with severe disease primarily in infants and older adults. The annual burden of RSV in adults in the United States is high, with an estimated 600,000–1,000,000 medically attended visits,^1^, ^2^, ^3^ 130,000–177,000 hospitalizations,^3^, ^4^ and 11,000–17,000 deaths^3^, ^5^, ^6^, ^7^, ^8^ resulting in substantial healthcare resource utilization and cost.

In the United States, hospitalization costs associated with RSV infection in adults are estimated to be between one and five billion dollars annually.^1^, ^3^ Available hospitalization cost data have been primarily derived by extrapolating costs from administrative databases, or by using influenza and pneumonia cost data to estimate costs for RSV.^1^, ^3^, ^5^ In one study utilizing national administrative databases to compare RSV‐associated hospitalization costs with influenza‐ and unspecified viral pneumonia‐associated hospitalization costs, the average length of stay (LOS) was longer for RSV hospitalizations, and the mean adjusted cost for RSV hospitalization ($38,828) was more than double than that for influenza ($14,519) or unspecified viral pneumonia ($18,051).^1^ Cost data from hospital administrative database studies are not optimal because RSV testing in adults is often incomplete, thereby resulting in underdiagnosis. Datta *et al* found that discharge diagnoses in adults underestimated RSV‐associated hospitalization by as much as 50%.^6^

Patient‐level cost data from prospective studies in which all suspect RSV cases are tested and ascertainment of RSV is complete could reduce bias and improve generalizability, but there are no published data using this approach. We designed a nested study within a large, prospective, multi‐center, multi‐season, population‐based, active surveillance study of RSV‐associated hospitalization in adults ≥ 18 years of age^4^ and collected epidemiologic and cost data, to estimate the cost of RSV‐associated hospitalizations and determine factors associated with higher costs.

## 2 METHODS

### 2.1 Study site and population

Prospective surveillance for RSV infection was conducted over three RSV seasons at three hospital systems: NewYork‐Presbyterian Hospital, Columbia University Irving Medical Center (NYP‐CUIMC) in New York City; the University of Rochester Medical Center ‐ Strong Memorial Hospital (URMC); and Rochester Regional Health System, Rochester General Hospital (RGH) in Rochester, NY.^4^ Patients included in this cost study were those included in the prospective surveillance study during the first two RSV seasons: October 15, 2017 to April 30, 2018 and October 15, 2018 to April 30, 2019. Informed consent to access cost data was required at the Rochester sites for inclusion in the study.

To identify patients with laboratory‐confirmed RSV infection, study staff reviewed infection control databases and clinical virology laboratory logs to ascertain the results of PCR tests ordered as standard of care for patients admitted with acute respiratory illness (ARI). Additionally, during periods of active surveillance, study staff reviewed admission logs and/or emergency department logs to identify patients meeting the screening case definition that included more than or equal to two ARI symptoms, or patients admitted with congestive heart failure (CHF), chronic obstructive pulmonary disease (COPD), or asthma preceded by ARI symptoms within the past 14 days. ARI was defined as two or more of the following criteria: presence of fever (≥37.8°C) or feeling feverish, new or worsening cough, new or worsening sputum production, new or worsening dyspnea, or sore throat, runny nose/nasal congestion, and/or body aches. For those who met the screening case definition, but were not tested for RSV by treating clinicians, the patient or their legally authorized representative was approached to obtain written informed consent for RSV testing in the research laboratory. As a further check for patients with RSV missed by active surveillance, when the surveillance seasons ended, the electronic medical record (EMR) was queried for all positive RSV tests in hospitalized adults during the study period. Eligible patients were adults ≥ 18 years of age with laboratory‐confirmed RSV who were hospitalized for ≥24 h and met the screening case definition. Patients with healthcare‐associated RSV infections, defined as RSV detected ≥3 days after admission, were not included in the study.^4^

### 2.2 Cost calculations

The primary outcome for this study was the cost of RSV‐associated hospitalization from admission to discharge or in‐hospital death. We extracted costs and/or charges from hospital billing and financial databases. For two of the three hospital systems, costs were directly provided. For the remaining hospital system, costs were estimated using inpatient charges. Charges were multiplied by the hospital's publicly available cost‐to‐charge ratio (CCR) from the 2018 Hospital Cost Report Data file maintained by the Centers for Medicare & Medicaid Services.^9^ We updated all costs to 2020 US dollars using the medical care component of the consumer price index.^10^

We determined total costs by combining direct costs of patient care (e.g., nursing, room and board, medications, and supplies) and overhead costs (e.g., administrative expenses to comply with federal and state regulatory requirements and maintain medical records).^11^ We did not include patients' productivity loss (e.g., loss of working hours) nor physician or professional fees as they were not captured in hospital discharge cost datasets.^12^ We described the costs attributed to the following categories set by the hospitals: Emergency Department (e.g., ED care prior to inpatient admission), nursing (e.g., bed in non‐ICU ward), pharmacy (medications administered), laboratory (laboratory tests and processing), therapy (e.g., respiratory interventions such as mechanical ventilation), ICU (bed in ICU), radiology (imaging studies), and other (e.g., endoscopy, dialysis, and echocardiography).

### 2.3 Cost predictors

Potential predictors of higher costs were abstracted from the medical record and included patients' demographic characteristics (age, sex, and pre‐admission living situation); type and number of comorbid conditions (respiratory, cardiac, immunosuppressive, or neurologic conditions, kidney or liver disease, diabetes mellitus, or obesity); Systemic Inflammatory Response Syndrome (SIRS) at presentation (defined as two or more of the following four criteria: temperature > 38.0°C or <36.0°C, tachycardia > 90 beats/minute, tachypnea > 20 breaths/minute, leukocytosis > 12 × 10^9^/L or leukopenia < 4 × 10^9^/L^13^, ^14^); healthcare resource utilization (LOS, ICU admission, ICU LOS, ventilator use, ventilator‐days, and antibiotic use); study site; and RSV season.

### 2.4 Data analyses

Continuous variables (e.g., LOS) were collapsed into multi‐level categorical variables. We evaluated the distribution of continuous variables in predicting costs and categorized each increment into the same bin when the association was similar.

We conducted univariate analyses to examine crude associations between predictors and costs. We also evaluated the distribution of LOS and ICU admission by predictors to associate costs with these healthcare resource utilization indicators. We compared mean costs and LOS against a pre‐defined reference level (e.g., no comorbidities) using the Wilcoxon signed‐rank test. Categorical variables were compared using chi‐squared or Fisher's exact test, as appropriate. In addition, we estimated empirical 95% confidence intervals (CI_95_) for LOS and costs by generating 10,000 bootstrap samples, recalculating means, and using the 2.5 and 97.5 percentiles from this sample.^15^ Finally, we described the distribution of cost in median value and interquartile range.

Cost predictors such as ICU admission and ventilator use can identify the same patients. To avoid multi‐collinearity in regression analysis, we conducted a cluster analysis that was set to split a cluster^16^ until the minimum proportion of variance explained by the cluster component was 75% of the total variance. Among each cluster, we chose the factor with the strongest predictive properties for high costs; for example, ICU admission was chosen from the cluster of ICU admission, ICU LOS, ventilator use, and ventilator‐days.

The conceptual framework for our study was to determine a set of predictors (patient attributes and clinical characteristics and outcomes) of higher hospitalization cost. The reason why we included clinical outcomes that occur during hospitalization such as LOS was to allow us to consider the effects of other predictors while controlling for the effects of these clinical outcomes in the cost model. ICU admission was a very strong predictor of cost, yet a small minority of patients was admitted to the ICU. Building two separate prediction models, one for patients admitted to the ICU and one for patients not admitted to the ICU, allowed us to estimate the effect of predictors on the cost that can vary by ICU admission. For each model, we manually removed correlated variables and those with weak statistical significance (*p* > 0.10) for which clinical relevance is also limited. We used multivariate generalized linear regression model (GLM), with a gamma cost distribution and a LOG link function to determine predictors of the cost of hospitalization. We used exponentiated beta coefficients (eβ) calculated from multivariate GLM to generate the cost ratios by predictors.

To estimate US annual cost, we extrapolated the age group‐specific mean costs by multiplying it by population‐based incidence from the prospective surveillance study^4^ and the size of the US adult population in 2019.^17^ We calculated CI for the extrapolated cost using the delta method.^18^

Finally, as a sensitivity analysis, we repeated univariate and multivariate data analyses after limiting the study cohort to only those who were alive at discharge.

All statistical analyses were conducted using SAS version 9.4. This study was approved by the Columbia University Medical Center and University of Rochester institutional review and privacy boards.

## 3 RESULTS

### 3.1 Study population

During the 2017–2018 and 2018–2019 RSV seasons, a total of 9774 patients were screened for eligibility. Of the 7120 patients who met the screening case definition, 6621 (93%) were tested for RSV. Overall, 756 patients with laboratory‐confirmed RSV were identified by combining cases identified by active and passive surveillance. Cost data were available for 79% (601/756) of patients hospitalized with laboratory‐confirmed RSV; these 601 patients were included in the current study. The mean age of these patients was 67 years (CI_95_ 65–68), approximately half of the patients lived independently in the community (46%), and the majority (95%) had at least one comorbidity, most commonly cardiovascular conditions (56%) (Table 1).

**TABLE 1:** Distribution of observed hospital cost, length of stay, and ICU admission stratified by predictors in adult patients with RSV‐associated hospitalizations

Predictors		*N* = 601	Costs (2020 $)	LOS (days)	ICU admission
		*n*, %	Mean [CI_95_]	Mean [CI_95_]	%
Age (years)					
Mean: 67 [CI_95_ 65–68]					
18–49 (reference)		85 (14%)	$11,124 [$6522–$17,386]	10 [6–14]	22%
50–64		173 (29%)	$7384 [$5743–$9534]	6 [5–7]	11%*
≥65		343 (57%)	$8241 [$6957–$9758]	8 [7–9]	15%
Gender					
Male (reference)		249 (41%)	$8763 [$7003–$10,893]	7 [6–9]	16%
Female		352 (59%)	$8148 [$6669–$9943]	8 [7–9]	14%
Comorbidities (reference: No)					
Chronic lung	Yes	296 (49%)	$7672 [$6552–$8968]	7 [6–8]	17%
	No	305 (51%)	$9111 [$7157–$11,483]	8 [7–9]	13%
Cardiovascular	Yes	337 (56%)	$9707* [$7827–$11,896]	8 [7–9]	15%
	No	264 (44%)	$6738 [$5673–$7983]	7 [6–9]	14%
Immunosuppression	Yes	174 (29%)	$8359 [$6760–$10,222]	8 [6–9]	13%
	No	427 (71%)	$8420 [$6949–$10,169]	8 [7–8]	16%
Neurologic	Yes	145 (29%)	$6824* [$5025–$9429]	7 [6–8]	10%
	No	456 (76%)	$8904 [$7533–$10,506]	8 [7–9]	16%
Diabetes mellitus	Yes	257 (43%)	$7596 [$6386–$9068]	7 [7–8]	15%
	No	344 (57%)	$9006 [$7223–$11,137]	8 [7–9]	14%
Obesity	Yes	179 (30%)	$7810 [$5903–$10,630]	7 [6–8]	12%
	No	422 (70%)	$8653 [$7287–$10,199]	8 [7–9]	16%
Chronic kidney disease	Yes	168 (28%)	$9022 [$7036–$11,370]	8 [7–10]	15%
	No	433 (72%)	$8163 [$6799–$9825]	7 [6–8]	15%
Chronic liver disease	Yes	42 (7%)	$13,422 [$7129–$21,471]	9 [6–13]	16%
	No	559 (93%)	$8025 [$6913–$9340]	7 [7–8]	15%
Comorbid condition count					
0 (reference)		33 (5%)	$6501 [$3303–$11,620]	9 [4–16]	24%
1–3		470 (78%)	$8359* [$7069–$9892]	8* [7–8]	15%
≥4		98 (16%)	$9250* [$6459–$12,848]	7 [6–9]	12%
Living situation on admission					
Independent (reference)		277 (46%)	$8609 [$6761–$10,901]	7 [6–8]	15%
At home with assistance of friends, family, or aide		245 (41%)	$7105 [$5661–$8970]	7 [6–8]	10%
Skilled nursing facility/assisted living		79 (13%)	$11,706* [$8694–$15,199]	12* [9–15]	27%*
RSV season					
2017–2018 (reference)		287 (48%)	$9375 [$7557–$11,518]	8* [7–9]	16%
2018–2019		314 (52%)	$7160 [$6182–$9286]	7 [6–8]	14%
Met SIRS criteria					
Yes		265 (44%)	$10,025* [$8205–$12,165]	8* [7–10]	21%*
No (reference)		336 (56%)	$7123 [$5751–$8892]	7 [6–8]	10%
LOS (days)					
Mean: 8 [CI_95_ 7–8]					
1–3 (reference)		158 (26%)	$2815 [$2473–$3197]	NA	4%
4–6		214 (36%)	$4874* [$3948–$6406]	NA	7%
7–10		127 (21%)	$8367* [$6817–$11,033]	NA	15%*
≥11		102 (17%)	$24,485* [$19,945–$30,066]	NA	47%*
Admitted to ICU					
Yes		88 (15%)	$20,577* [$15,909–$26,623]	16* [13–20]	NA
No (reference)		513 (85%)	$6313 [$5367–$7455]	6 [6–7]	NA
ICU LOS (days)					
Mean: 9 [CI_95_ 7–11]					
0 (reference)		515 (86%)	$6319 [$5378–$7456]	6 [6–7]	NA
1–2		16 (3%)	$7010 [$4204–$11,008]	6 [4–9]	NA
≥3		70 (12%)	$24,024* [$18,537–$31,310]	18* [15–23]	NA
Mechanical ventilation					
Yes		62 (10%)	$24,074* [$17,936–$32,209]	18* [14–23]	NA
No (reference)		539 (90%)	$6599 [$5662–$7694]	6 [6–7]	NA
Ventilator‐days^a^					
Mean: 11 days [CI_95_ 8–14]					
0 (reference)		523 (87%)	$6421 [$5478–$7545]	6 [6–7]	NA
1–7		30 (7%)	$12,534* [$9568–$15,890]	10* [8–13]	NA
≥8		31 (6%)	$33,505* [$23,740–$47,157]	25* [19–33]	NA
Antibiotic use					
Yes		459 (76%)	$8883* [$7594–$10,424]	8* [8–9]	18%*
No (reference)		142 (24%)	$6852 [$4659–$9828]	5 [4–6]	4%
Died during hospitalization					
Yes (reference)		27 (4%)	$8108* [$6936–$9468]	12 [9–17]	63%
No		574 (96%)	$14,669 [$9140–$21,350]	7* [7–8]	37%*

Abbreviations: CI, confidence interval; ICU, intensive care unit; LOS, length of stay; NA, not applicable; RSV, respiratory syncytial virus; SIRS, Systemic Inflammatory Response Syndrome.

^a^ Ventilator‐days were unavailable for one patient.

* *p* < 0.05 when compared against reference.

### 3.2 Distribution of healthcare resource utilization and costs

The mean hospital LOS was 8 days (CI_95_ [7–8]). Overall, 15% of patients were admitted to the ICU (mean ICU LOS 9 days (CI_95_ [7–11]), 10% of patients required mechanical ventilation (mean 11 days [CI_95_ 8–14]), and 4% died during hospitalization (Table 1).

The mean total cost of hospitalization was $8403 (CI_95_ $7240–$9741). The mean costs per category were as follows: ICU $7885 (CI_95_ $5877–$10 240), nursing $4081 (CI_95_ $3573–$4644), pharmacy $1258 (CI_95_ $708–$1999), therapy $653 (CI_95_ $470–$912), emergency department $649 (CI_95_ $615–$683), laboratory $619 (CI_95_ $561–$684), radiology $324 (CI_95_ $275–$376), and other $630 (CI_95_ $482–$807). The median total cost of hospitalization was $4399 (interquartile range $2382–$8325) and distribution by cost category and predictor is reported in the supporting information.

### 3.3 Predictors of RSV hospitalization cost

In univariate analyses, predictors for higher costs included cardiovascular comorbidities, greater than or equal to four comorbidities, living in a skilled nursing facility at admission, meeting the SIRS criteria at presentation, hospital LOS ≥ 4 days, ICU admission, ICU LOS ≥ 3 days, mechanical ventilation, ventilator‐days ≥ 1, use of antibiotics, and in‐hospital mortality (Table 1). Most cost predictors were significantly associated with longer LOS and/or ICU admission.

In the multivariate model for patients *without* ICU admission, predictors of higher cost included LOS ≥ 4 days and chronic liver disease (Table 2) after adjusting for other comorbidities and clinical characteristics. Costs were two to nine times higher for patients with LOS ≥ 4 days compared with patients with LOS 1–3 days. Costs for patients with chronic liver disease were 38% higher compared with those without chronic liver disease.

**TABLE 2:** Multivariate predictors associated with higher RSV hospitalization costs in patients *without* and *with* ICU admission

Predictors	WITHOUT ICU admission		WITH ICU admission	
	Cost ratio	CI_95_	Cost ratio	CI_95_
RSV season 2018–2019 versus 2017–2018	0.89	(0.77–1.02)	1.29	(0.94–1.77)
Age (reference: 18–49 years)				
50–64	0.98	(0.76–1.27)	0.66	(0.38–1.12)
≥65	0.87	(0.72–1.06)	0.56	(0.33–0.95)*
Living situation (reference: independent)				
At home with help of friends, family, or aide	1.02	(0.88–1.19)	1.08	(0.80–1.44)
Skilled nursing facility/assisted living	0.85	(0.69–1.06)	1.06	(0.79–1.42)
LOS (reference: 1–3 days)				
4–6	2.08	(1.77–2.45)*	1.34	(0.76‐2.37)
7–10	3.82	(3.20–4.56)*	2.43	(1.41‐4.20)*
≥11	9.23	(7.15‐11.91)*	8.23	(4.48‐15.10)*
Comorbidities (reference: not present)				
Lung disease	0.91	(0.78–1.08)	0.82	(0.65–1.03)
Cardiovascular disease	1.12	(0.99–1.27)	1.08	(0.78–1.48)
Chronic liver disease	1.38	(1.05–1.80)*	1.21	(0.83‐1.76)
Immunosuppression	1.41	(0.98–1.33)	0.90	(0.66–1.23)
Neurologic conditions	0.97	(0.83–1.13)	1.07	(0.79–1.47)
Comorbid condition count (reference: 0)				
1–3	1.12	(0.93–1.36)	1.17	(0.67–2.04)
≥4	1.06	(0.82–1.38)	1.17	(0.55–2.50
Met SIRS criteria (reference: not met)	1.01	(0.83–1.22)	0.90	(0.71–1.13)
Antibiotic use (reference: not used)	0.91	(0.72–1.16)	1.49	(1.10–2.03)*

Abbreviations: ICU, intensive care unit; LOS, length of stay; RSV, respiratory syncytial virus; SIRS, Systemic Inflammatory Response Syndrome.

* *p* < 0.05 when compared against reference.

In the multivariate model for patients *with* ICU admission, age 18–49 years, LOS, and antibiotic use were significantly associated with higher costs (Table 2). Costs for patients aged 18–49 years were 79% higher compared with patients aged ≥ 65 years. Costs were two to eight times higher for patients with LOS ≥ 7 days compared with patients with LOS 1–3 days. Costs for patients treated with antibiotics were 49% higher compared with those not treated with antibiotics.

### 3.4 Nationwide RSV hospitalization costs

We used the mean total cost of hospitalization to extrapolate the annual US RSV‐associated hospitalization in adults and estimated this cost to be $1.2 (CI_95_ $0.9–$1.4) billion (Table 3).

**TABLE 3:** Extrapolated nationwide respiratory syncytial virus (RSV) hospitalization costs in adults in the United States

Age group (years)	Mean cost (2020 $) per hospitalization and CI_95_	Incidence rate per 100,000 persons and CI_95_ ^a^, ^4^	US population estimates as of 2019^19^	Total and CI_95_ ^b^
18–49	$11,124 [$6522–$17,386]	9.8 [7.8–12.3]	138,216,422	$150,676,909 [$42,505,143–$258,848,674]
50–64	$7384 [$5743–$9534]	57.3 [49.2–66.9]	62,925,688	$266,240,600 [$156,774,727–$375,706,472]
>65	$8241 [$6957–$9758]	167 [150.9–184.7]	54,058,263	$743,975,223 [$542,253,355–$945,697,091]
Grand total				$1,160,925,844 [$907,222,838–$1,414,628,849]

^a^ Standard errors were estimated by using the delta method for which the correlation measurement was assumed to be 1.

^b^ Limited to 2017–2018 and 2018–2019 RSV season data to be consistent with the current study.

### 3.5 Sensitivity analysis of costs among those surviving to discharge

We conducted a sensitivity analysis by excluding 27 patients (4%) who died during hospitalization as those who died had significantly higher costs than surviving patients ($13,741, vs. $7579, Table 1). The sensitivity analysis identified the same cost predictors associated with high costs for surviving patients in both the non‐ICU and ICU multivariable models (data not shown).

## 4 DISCUSSION

To our knowledge, this is the first study to evaluate individual patient‐level direct costs associated with laboratory‐confirmed RSV hospitalization identified through active surveillance. The total mean cost of hospitalization was $8403; costs were significantly higher ($20,577) for the 15% of patients admitted to the ICU. Previous studies describing costs of RSV‐associated hospitalization in adults reported costs higher than ours.^1^, ^20^, ^21^ Ackerson *et al* recently reported a mean cost of $15,163 (2013 USD) for adults aged ≥ 60 years hospitalized with RSV.^20^ Pastula *et al* reported a mean cost of $38,828 (2015 USD) for adults aged ≥ 20 years hospitalized with RSV.^1^ Finally, Han *et al* estimated a mean cost of $11,000 (1999 USD) for adults aged ≥ 65 years hospitalized with RSV‐associated pneumonia.^21^

Differences in methodology for case selection and cost calculations across studies could explain why our cost estimates were relatively low. None of the previous studies conducted active surveillance and most relied on diagnostic codes (e.g., ICD‐10‐CM, International Classification of Diseases, Tenth Revision, Clinical Modification) to identify RSV cases. In a recent analysis of adults with laboratory‐confirmed RSV infection seen in the emergency department or hospital, only 6% of medical records listed RSV as the primary discharge diagnosis, and only 51% included RSV in any discharge diagnosis.^6^ It is possible that if RSV testing was selectively performed on sicker adult patients who are likely to progress to more severe outcomes, as has been noted for infants,^22^ the cost of hospitalization could be overestimated.

Among others, Ackerson *et al* used an approach to identify RSV cases that was most similar to the methods used in our study by limiting cases to laboratory‐confirmed RSV, not relying on diagnostic codes, nor limiting the study population to certain conditions (e.g., pneumonia).^20^ Their mean LOS for adults aged ≥ 60 years was 7.58 days (CI_95_ 7.02–8.16), similar to that for the adults aged ≥ 65 years in our study (8 days, CI_95_ 7–9). However, their mean cost adjusted to 2020 USD, $19,871 ($18,198–21,613), were more than twice that estimated in our study. One difference might be that we used patient‐level charge or cost data directly from administrative records while Ackerson *et al* extrapolated the cost from the 2013 National Inpatient Sample by multiplying the LOS of laboratory‐confirmed RSV cases by the daily average hospitalization cost per diagnosis‐related group (DRG). The assignment to DRGs was based on the primary or secondary diagnoses and procedures during the hospital stay. Because RSV is not often coded as a primary diagnosis, having a chronic underlying medical condition or complication as the primary diagnosis,^6^ could lead to DRG‐based costs being overestimated. Finally, our study excluded healthcare‐associated RSV for which testing was ordered >72 h after admission. Studies that relied on diagnostic codes may have included healthcare‐acquired RSV which has been demonstrated to be more costly than community‐acquired RSV.^23^

We found that most predictors of high cost of RSV hospitalizations reflected greater healthcare resource use (i.e., longer hospital stay or ICU admission) and/or more complex hospital course (i.e., antibiotic use). For patients without ICU admission, chronic liver disease was identified as an additional predictor; the reason for increased cost in patients with chronic liver disease was uncertain because their cost breakdowns (e.g., pharmacy) were similar to patients without chronic liver disease, except for higher nursing costs. For patients with ICU admission (15%), unique predictors for high costs included age 18–49 years and antibiotic use. The finding that younger age is associated with higher cost may seem unexpected, because older age is clearly a risk for RSV‐associated hospitalization. However, this may reflect that younger adults who were hospitalized with RSV could be much sicker (e.g., more complex comorbidities or hospital course) as evidenced by longer LOS compared with older adults. Because the cost predictors identified in this study are not easily preventable, development of prevention strategies, including vaccines, for RSV infections in adults may be useful in reducing RSV infections, hospitalizations, and subsequent healthcare costs.

Over the first two seasons of the RSV prospective surveillance study, 9774 patients were screened for eligibility, of whom 7120 met the case definition for RSV testing, and 6621 (93%) were tested for RSV.^4^ Using these robust incidence data, along with patient‐level cost data, we extrapolated the total cost of RSV hospitalizations in the United States to be an estimated $1.2 billion dollars annually. This national, annual burden estimate should be taken as a conservative estimate, as we did not include physician fees; costs of continued care (e.g., long‐term sequalae or exacerbations of chronic conditions); nor productivity loss experienced by patients and/or caregivers.

Our study has several strengths and limitations. The main strength of our study was the ability to reduce outcome misclassification by conducting active surveillance and systematically identifying patients with laboratory‐confirmed RSV infection. An additional strength was the availability of patient‐level cost and/or charge data. However, the study has several limitations. We were able to utilize only 80% of the first‐year data as not all subjects could be reached post‐discharge to obtain consent at the Rochester sites. However, we do not believe that this attrition introduced participation bias as the distribution of demographics and underlying medical conditions were similar between participants and non‐participants. Similar to previous studies,^1^, ^20^, ^21^ we did not include costs incurred from professional fees or physician charges that were outside the hospital charges.^12^ Although the omission of such professional fees and physician charges is common in analyses based on hospital discharge data,^17^, ^24^ we likely underestimated the full cost of hospitalization by 20%–25% based on previous estimates of these additional costs.^25^, ^26^ One hospital system calculated costs utilizing Centers for Medicare & Medicaid Services (CMS's) CCR, which can vary by cost center. Taking the average CCRs could bias cost estimates; however, the direction of the bias is unknown. Finally, our extrapolation to a national annual cost of RSV‐associated hospitalizations assumed non‐differential healthcare resource consumption in other regions of the United States. We would like to acknowledge that this estimate may not be generalizable to settings with substantially different patient demographics, healthcare practices, and unit cost of care.

## 5 CONCLUSION

This is the first study that evaluated the patient‐level cost of laboratory‐confirmed RSV‐associated hospitalization in adults identified through passive and active surveillance. Our data increase the accuracy of estimates of the mean total cost of RSV‐associated hospitalization and allow an extrapolation of the economic burden of RSV hospitalization in adults ≥ 18 years in the United States, estimated to be 1.2 billion dollars annually. It is possible that an adult RSV vaccination program could be useful in averting RSV‐associated healthcare resource use and cost.

## AUTHOR CONTRIBUTIONS

**Yoonyoung Choi:** Formal analysis; investigation; methodology; project administration; supervision. **Alexandra Hill‐Ricciuti:** Formal analysis; investigation. **Angela Branche:** Conceptualization; funding acquisition; investigation; supervision. **William Sieling:** Formal analysis; investigation. **Lisa Saiman:** Conceptualization; funding acquisition; investigation; supervision. **Edward Walsh:** Conceptualization; funding acquisition; investigation; supervision. **Matthew Phillips:** Conceptualization; project administration. **Ann Falsey:** Conceptualization; funding acquisition; investigation; supervision. **Lynn Finelli:** Conceptualization; investigation; methodology; supervision.

## FUNDING INFORMATION

Funding for this research was provided by Merck Sharp & Dohme Corp., a subsidiary of Merck & Co., Inc., Kenilworth, NJ, USA.

## Supporting information


**Table S1.** Distribution of observed hospital cost in median and IQR values
