# Supplementary material for: Cost determinants among adults hospitalized with respiratory syncytial virus in the United States, 2017–2019
Source: Influenza Other Respir Viruses. 2021 Oct 3;16(1):151–8. doi: 10.1111/irv.12912 (PMC8692803; doi:10.1111/irv.12912)
Supplement: Supplementary file 1 — Table S1. Distribution of observed hospital cost in median and IQR values [file IRV-16-151-s001.docx]

**Supplemental Material.**

**Table. Distribution of observed hospital cost in median and IQR values**

| **Category** | | **Costs $** |
| --- | --- | --- |
|  |  | **Median [IQR]** |
| **Total** | | $4,399 [$2,382-$8,325] |
| ICU | | $4,672 [$2,105-$10,272] |
| Nursing | | $2,567 [$1,262-$4,902] |
| Pharmacy | | $150 [$55-$497] |
| Therapy | | $168 [$72-$524] |
| Emergency Department | | $724 [$146-$837] |
| Laboratory | | $390 [$248-$642] |
| Radiology | | $107 [$55-$297] |
| Other | | $51 [$14-$399] |
| **Predictors** | | **Median [IQR]** |
| **Age** | | |
| 18-49 | | $4,078 [$2,233-$8,202] |
| 50-64 | | $4,399 [$2,707-$7,320] |
| >65 | | $4,530 [$2,286-$8,939] |
| **Gender** | | |
| Male | | $4,646 [$2,493-$8,960] |
| Female | | $4,215 [$2,339-$7,916] |
| **Comorbidities** | | |
| Chronic Lung | Yes | $4,483 [$2,560-$8,618] |
|  | No | $4,342 [$2,354-$8,080] |
| Cardiovascular | Yes | $4,981 [$2,752-$8,936] |
|  | No | $3,697 [$2,162-$7,409] |
| Immunosuppression | Yes | $4,760 [$2,952-$8,684] |
|  | No | $4,224 [$2,280-$8,325] |
| Neurologic | Yes | $3,712 [$2,157-$7,007] |
|  | No | $4,591 [$2,499-$8,910] |
| Diabetes mellitus | Yes | $4,778 [$2,402-$8,390] |
|  | No | $4,102 [$2,379-$8,193] |
| Obesity | Yes | $4,609 [$2,325-$8,080] |
|  | No | $4,296 [$2,382-$8,618] |
| Chronic Kidney Disease | Yes | $4,649 [$2,491-$8,381] |
|  | No | $4,213 [$2,354-$8,202] |
| Chronic Liver Disease | Yes | $6,300 [$3,231-$11,232] |
|  | No | $4,270 [$2,353-$8,148] |
| **Comorbid Condition Count** | | |
| 0 | | $3,237 [$2,093-$4,477] |
| 1-3 | | $4,444 [$2,325-$8,431] |
| >4 | | $4,683 [$3,166-$8,317] |
| **Living Situation on Admission** | | |
| Independent | | $4,092 [$2,325-$8,184] |
| At home with assistance of friends, family, or aide | | $4,181 [$2,311-$7,152] |
| Skilled nursing facility/assisted living | | $6,600 [$3,166-$14,759] |
| **RSV Season** | | |
| 2017-2018 | | $4,530 [$2,461-$8,900] |
| 2018-2019 | | $4,264 [$2,280-$7,943] |
| **Met SIRS criteria** | | |
| Yes | | $4,933 [$2,702-$9,951] |
| No | | $3,976 [$2,223-$7,490] |
| **LOS** | | |
| 1-3 | | $2,019 [$1,220-$3,605] |
| 4-6 | | $3,274 [$2,354-$5,165] |
| 7-10 | | $6,548 [$4,794-$9,301] |
| >11 | | $17,021 [$10,198-$29,419] |
| **Admitted to ICU** | | |
| Yes | | $16,719 [$6,942-$23,874] |
| No | | $3,694 [$2,171-$6,943] |
| **ICU LOS** | | |
| 0 | | $3,700 [$2,171-$6,950] |
| 1-2 | | $4,559 [$3,589-$6,829] |
| >3 | | $17,730 [$9,653-$28,043] |
| **Mechanical Ventilation** | | |
| Yes | | $17,635 [$8,684-$30,139] |
| No | | $3,742 [$2,240-$7,124] |
| **Ventilator-days** | | |
| 0 | | $3,712 [$2,210-$6,994] |
| 1-7 | | $8,368 [$4,751-$17,586] |
| >8 | | $21,348 [$17,552-$39,069] |
| **Use of antibiotics** | | |
| Yes | | $4,727 [$2,493-$8,960] |
| No | | $3,697 [$2,172-$6,550] |
| **Died during hospitalization** | | |
| Yes | | $8,325 [$4,364-$19,094] |
| No | | $4,219 [$2,353-$8,102] |

IQR=Interquartile range; ICU=intensive care unit; LOS=length of stay; SIRS= Systemic Inflammatory Response Syndrome
